# Supplementary material for: Direct observation of vacuum arc evolution with nanosecond resolution
Source: Sci Rep. 2019 May 24;9:7814. doi: 10.1038/s41598-019-44191-6 (PMC6534580; doi:10.1038/s41598-019-44191-6)
Supplement: Supplementary file 1 — Supplementary to: Direct observation of vacuum arc evolution with nanosecond resolution [file 41598_2019_44191_MOESM1_ESM.pdf]

Supplementary to: Direct observation of vacuum arc evolution with nanosecond resolution

Zhipeng Zhou[1,2], Andreas Kyritsakis[2], Zhenxing Wang[1*], Yi Li[1], Yingsan Geng[1], and Flyura Djurabekova[2,3]

[1]State Key Laboratory of Electrical Insulation and Power Equipment, Xi'an Jiaotong University, Xi'an 710049, China
[2]Helsinki Institute of Physics and Department of Physics, University of Helsinki, P.O. Box 43, FI-00014 Helsinki, Finland
[3]National Research Nuclear University MEPhI, Kashirskoye sh. 31, 115409 Moscow, Russia
[*]zxwang@xjtu.edu.cn

S1 Determination of the breakdown instant

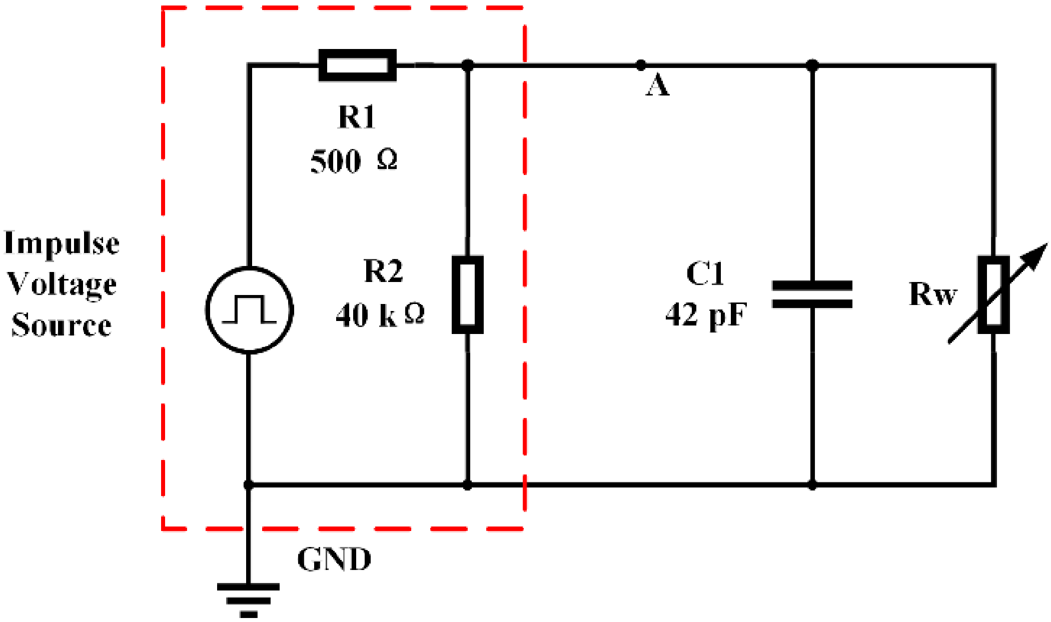

Figure S1. Schematic of the simplified circuit representing the vacuum arc system.

Figure S1 demonstrates a simplified circuit model of the experiments. The impulse voltage source consists of three components enclosed by a dash line rectangular, an ideal pulse voltage source, a current-limiting resistor R1 and a discharge resistor R2. R1 is 500 Ω, and R2 is 40 kΩ. The vacuum gap together with the chamber are represented by a capacitor and a variable resistor in parallel. The total capacitance of the system C1 was measured 42 pF. Rw varies from infinity before the breakdown (open circuit) to a very small value after the full breakdown to (short-circuit). The total current is measured using the current sensor on the wire between the high voltage terminal of the voltage source and the upper connector (connected to the cathode) of the chamber, i.e. at the point *A* shown in the schematic. The gap voltage *V* is measured at the same point, between the gap electrodes. Finally, the gap current is obtained by subtracting the capacitive component $C_1 dV/dt$ of the current flowing through C_1 from the total current as measured at *A*.

Figure S2(a) shows the waveform waveforms for the total current and the gap current of a vacuum breakdown. The electrode configuration is tip-to-plane with a gap length of 5 mm, and the voltage pulse width is 1 μs. The dashed line in Figure S2(a) represents the gap current, while the solid line represents the total current. By comparing the total current and the gap current, we can find a turning point, which is marked by a red dash circle, on the total current waveform at t_0, coinciding with the starting point of the current rise in the gap.

We shall prove now that the turning point in Figure S2(a) is the starting point of a vacuum breakdown. We simulated the circuit model of Figure S1 in Simulink. Rw was set initially to 100 MΩ and 100 ns after the application of the pulse from the

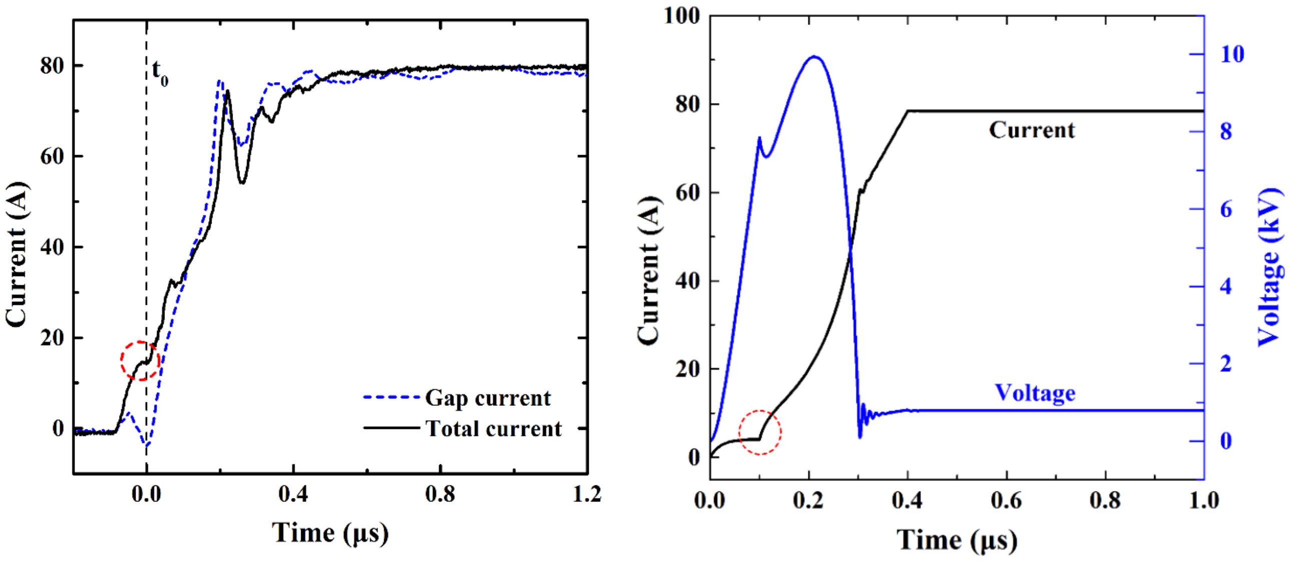

Figure S2. (a) Waveforms of the total current and the gap current during a vacuum breakdown. (b) Simulated waveforms of the total current and the gap voltage.

voltage source dropped instantly to 1000 Ω, to represent the initiation of phase P1. Then Rw gradually dropped to 10 Ω in the next 200 ns to simulate the ignition phase P1 of the breakdown. As a result, we obtain the current and voltage waveforms shown in Figure S2(b). The voltage and current waveforms are very similar to the experimental ones. Furthermore, a turning point marked by a dash circle appears exactly at 100 ns in Figure 1, indicating that the point is a result of the breakdown in the vacuum gap. Therefore, we can confirm that indeed the time point t_0 is the beginning of a vacuum breakdown. Finally, we note that the voltage keeps increasing after the breakdown instant, both in the experimental and the simulations curves.

S2 Verification for the repeatability of the experiments

The high voltage used in our experiments ensured the repeatability of the process with high accuracy. However, we verified this repeatability in three independent, but identical, experiments by comparing the current waveforms. We observed some variations in the shape of the waveforms; yet, the overall behaviour was found to be very similar (see Fig. S3(a)). In Fig. S3(b), we show the snapshots taken in these experiments, with an exposure time of 7 μs, that covers the whole breakdown process. All three snapshots look very similar displaying the same overall behaviour of the arc.

S3 Vacuum arc evolution for different pulse durations

Figure S4 demonstrates the current waveforms obtained for different voltage pulse durations, varying from 1 to 5 μs. The main figure demonstrates the rising edge of the currents focusing in phases P0 and P1, while the inset shows the corresponding complete current waveforms. The current pulse widths in the figure are consistent with the applied voltage pulse widths. Furthermore, the rising dynamics of all current waveforms are very similar. This allows us to conclude that the initial process of a vacuum breakdown is not affected by the voltage pulse width.

S4 Quantitative analysis of the total gap light

Figure S5 depicts the intensity of the cathode light emission for lengths varying from 0.5 mm to 5 mm and voltage pulse durations from 1 μs to 5 μs. The data were obtained by repeating the experiment 10 times for each case and calculating the corresponding average and the standard deviation for the error bars. Figure S5(a) depicts the value of the maximum intensity and S5(b) the value of the total integrated intensity, obtained by summing all the pixels of an image such as Figure 5(a) of the main text, i.e. with the camera focused at the cathode region. Table 1 gives the corresponding full-width-half maximum range of the peak shown in figure 5b of the main text.

As shown in the figure, both the maximum and the total intensities increase with the voltage pulse width, and the total intensities increase with the voltage pulse width in a roughly linear manner. For a specific voltage pulse width, however, they do not vary significantly. The above mean that the intensity of the cathode spot remains practically constant both for any gap

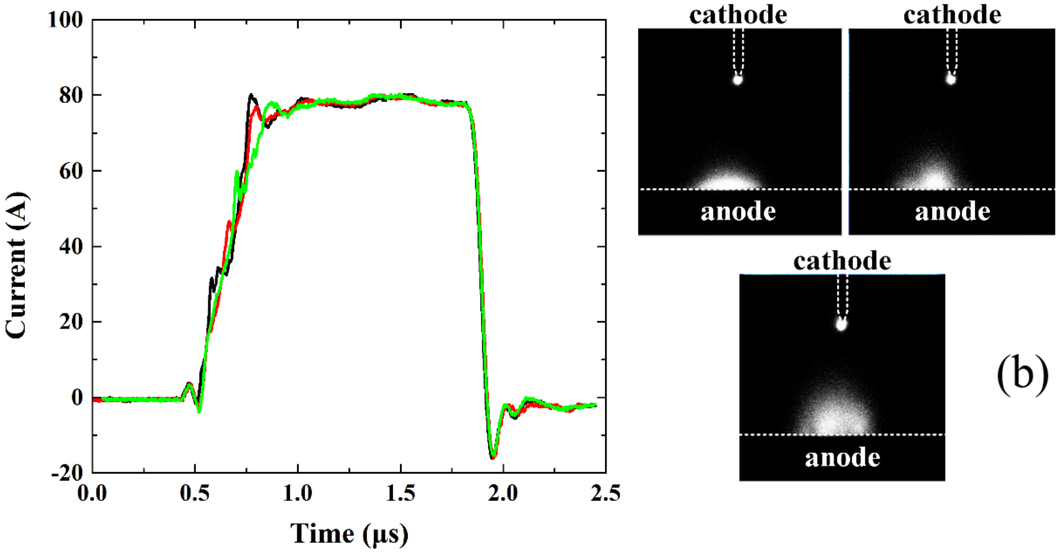

Figure S3. Repeatability test. Three independent, but identical experiments result in the same waveforms of the gap current (a). Some variation in the shapes can be observed, however the general behavor is very similar.(b) shows the three snapshots taken in these experiments with the high-speed shutter of the ICCD camera being open for 7 μs (entire arc).

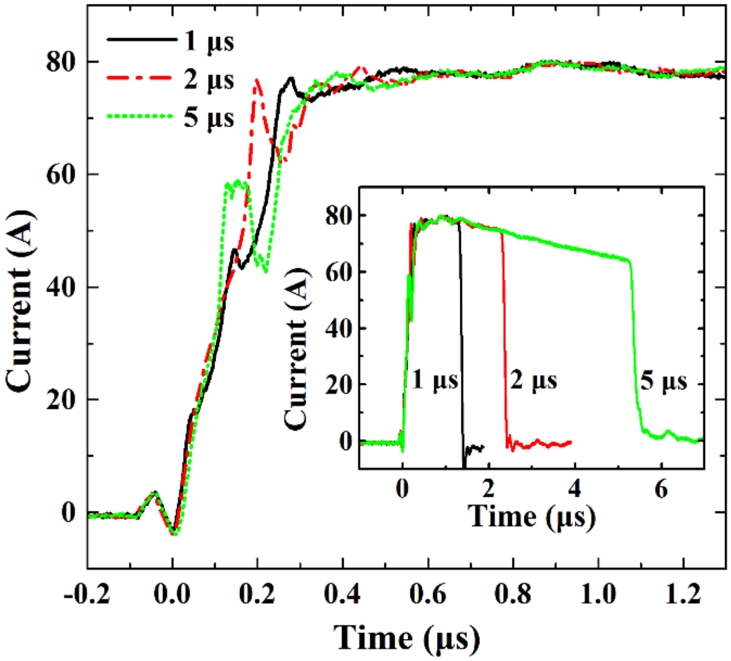

Figure S4. Current waveforms of vacuum breakdowns under different voltage pulse durations from 1 μs to 5 μs. The gap length is 5 mm.

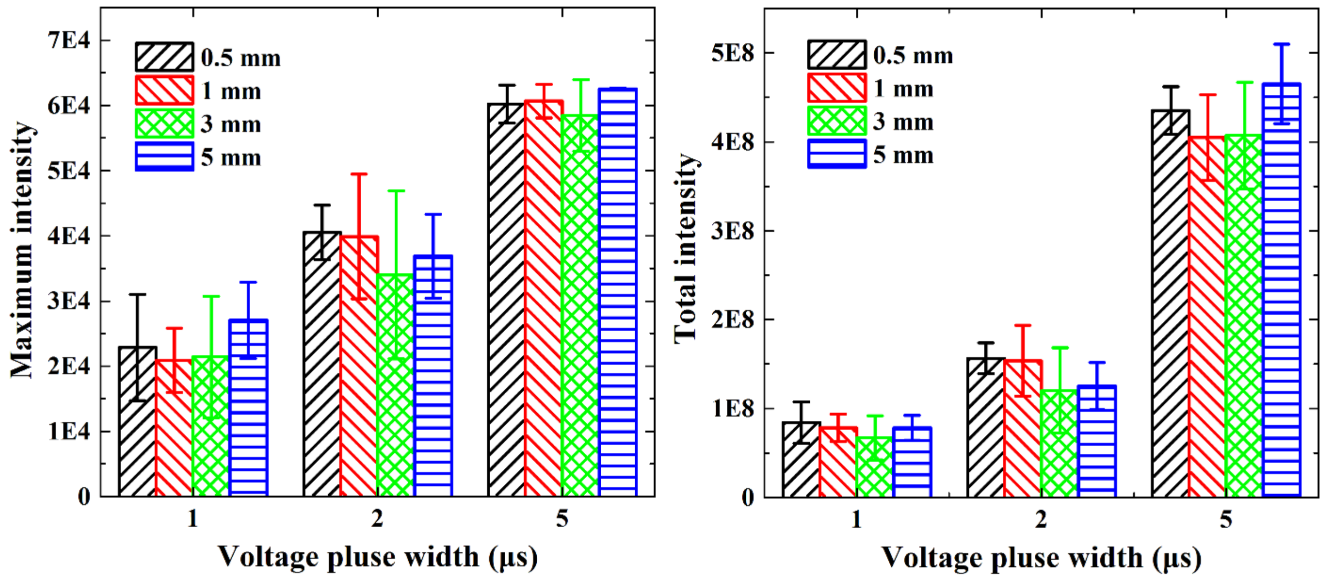

Figure S5. The maximum (a) and integrated total (b) intensity of the cathode light as a function of the voltage pulse duration and the gap length.

Gap length	FWHM mean value	FWHM std. deviation
5 mm	0.112 mm	0.011 mm
3 mm	0.115 mm	0.022 mm
1 mm	0.102 mm	0.022 mm
0.5mm	0.094 mm	0.019 mm

Table 1. Full width at the half maximum of the cathode spot peak for various gap lengths. Both the mean value and the standard deviation as obtained by 10 experimental repetitions are given.

length or pulse duration. The signal captured in the camera increases with the pulse duration only because the exposure time increases respectively.

S5 Space charge calculations

Any type of electron emission – field, thermionic or mixed – is limited by the space charge. In other words, if the emitted current density exceeds a certain limit, the escape rate of the electrons from the surface become comparable to their cathode-anode flight time. The forming cloud of emitted electrons creates a significant space charge in the vacuum above the emitting surface that screens the effect of applied field. In turn, this naturally limits the current density. This phenomenon, known as a space charge can give an accurate estimate of the maximum current density J_c, which is possible for a specific geometry and experimental condition. For this, we use the Child-Langmuir law[1]

$$J_c = \frac{4}{9\kappa}\frac{F^2}{V^{1/2}}, \kappa = \frac{m^{1/2}}{\varepsilon_0 (2e)^{1/2}} \tag{1}$$

where V is the applied voltage and F is the magnitude of the local field, calculated without taking into account the space charge. We note that the Child-Langmuir law is developed for planar geometries, however particle-in-cell simulations have shown that it is a valid approximation for surfaces that have radii of curvature of the order of several μm[2]. Here we consider only the magnitude of the field and of the current density, since their directions at least in the vicinity to the surface coincide and are always perpendicular to the emitting surface.

At every point r at the cathode, F is proportional to V, i.e. $F(r) = \xi(r)V$. In the latter relation, ξ is the local voltage-to-field conversion factor, which is measured in units [1/m]. Substituting to eq. (1) yields:

$$J_c(r) = \frac{4}{9\kappa}\xi^2(r)V^{3/2} \tag{2}$$

Given the distribution of $\xi(r)$ on the emitting cathode surface S, the maximum space-charge-limited total current can be obtained as a function of the applied voltage by integrating the current density over the emitting area S:

$$I_c = \int_S J_c dA = \frac{4}{9\kappa}V^{3/2}\int_S \xi^2 dA = \frac{4}{9\kappa}V^{3/2}\zeta \tag{3}$$

where ζ is a dimensionless parameter that depends on the geometry. In the above equation, we consider the current density to be perpendicular to the surface and therefore the vector surface integral simplifies to a scalar one. In the final formula giving I_c, ζ represents the total contribution of the geometry. To give an intuitive understanding of its value, if our cathode was planar, it would be $\zeta = A/d^2$ where A would be the total area of the cathode electrode.

The distribution $\xi(r)$ is calculated according to the finite element method described in the method section of the main text. Table 2 contains the calculated maximum apex values of ξ and the corresponding values of ζ as obtained by numerically integrating eq. (3).

d	ζ	ξ_{max}
5 mm	2.25	10.9 mm^{-1}
3 mm	2.65	11.7 mm^{-1}
1 mm	3.71	13.9 mm^{-1}
0.5mm	5.16	16.4 mm^{-1}

Table 2. Calculated ζ and ξ_{max} for various gap distances.

In Figure S6 we show the voltage waveform $V(t)$ (the left ordinate) through the gap with the standard tip-to-plane electrode configuration and the gap length 5 mm. The measured current waveform through the gap $I(t)$ is shown in the red curve (right ordinate), and the corresponding theoretical estimation of the maximum space-charge limited current $I_c(t)$ obtained by eq. (3), assuming the same $V(t)$ as in the experiment is shown in the green curve. The camera images taken at the 50 ns intervals are placed at the top of the graph and matched with the corresponding intervals in the horizontal axis.

We clearly see that the measured current significantly exceeds the space-charge limit long before the anodic glow begins. We observe the same feature for all gap distances 1 mm, 3 mm, 5 mm. The high values of the measured current suggest that these are rather discharge currents, and not pure electron emission currents. During the discharge, the forming plasma compensates the vacuum space charge, and the currents extracted from the surface rise much higher than those due to a direct electron emission process.

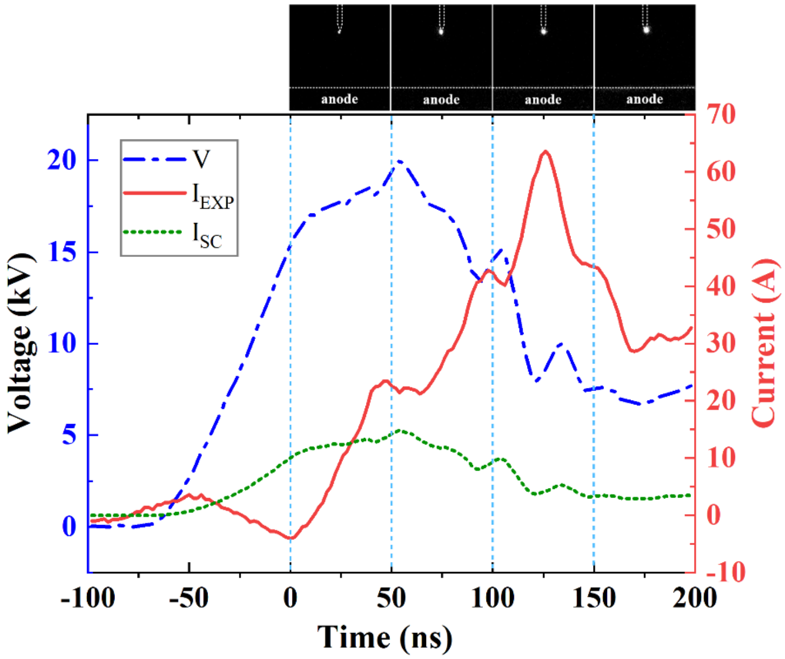

Figure S6. Voltage (blue line, left axis) and gap current (red line, right axis) waveforms measured in the default tip-to-plane configuration with the 5 mm gap length. Green dash line shows the calculated maximum space-charge limited gap current. $I_{sc}(t)$ was calculated assuming the experimental $V(t)$ waveform.

S6 Additional arc image sequences

Figures S7 and S8 show the captured image sequences for gap distances of 1 and 3 mm respectively.

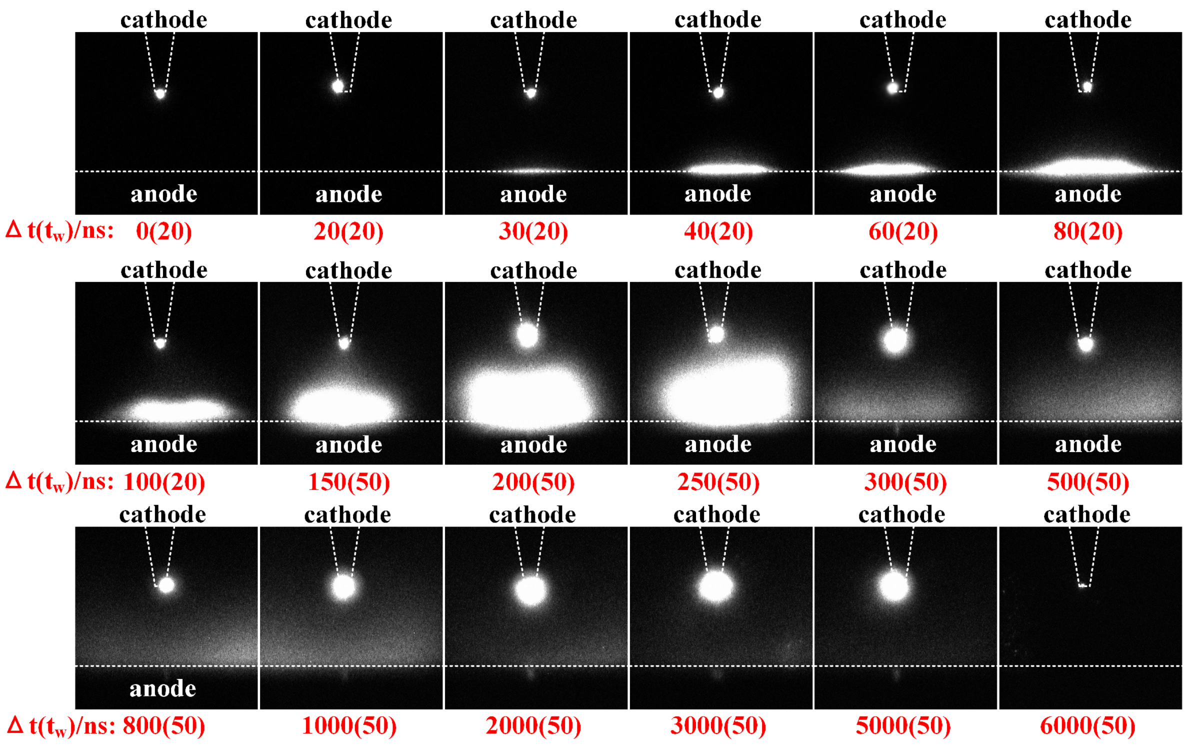

Figure S7. Arc image sequences in the gap as captured by the ICCD camera with various delay times for a gap distance of 1 mm.

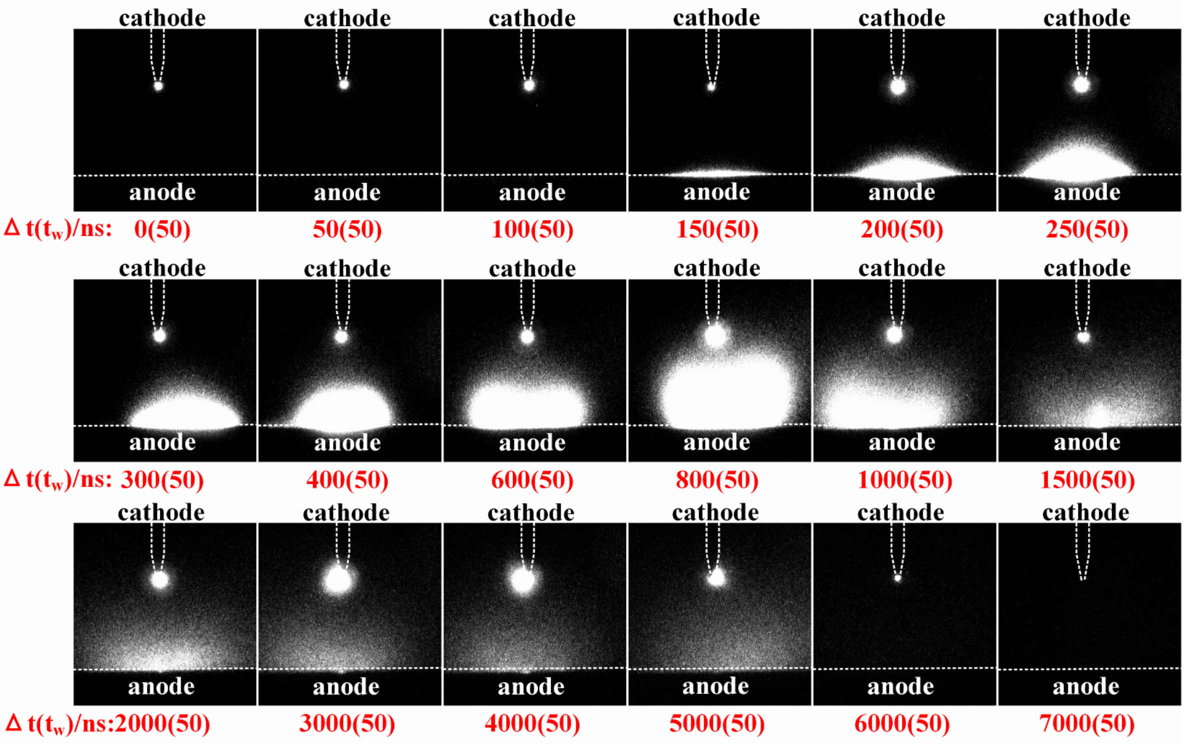

Figure S8. Arc image sequences for a gap distance of 3 mm.

References

1. Child, C. D. Discharge from hot cao. *Phys. Rev. (Series I)* **32**, 492–511 (1911). DOI 10.1103/PhysRevSeriesI.32.492.

2. Uimanov, I. V. The dimensional effect of the space charge on the self-consistent electric field at the cathode surface. *IEEE Transactions on Dielectr. Electr. Insulation* **18**, 924–928 (2011). DOI 10.1109/TDEI.2011.5931082.
